# Coding and long non-coding RNAs provide evidence of distinct transcriptional reprogramming for two ecotypes of the extremophile plant *Eutrema salsugineum* undergoing water deficit stress

**DOI:** 10.1186/s12864-020-06793-7

**Published:** 2020-06-08

**Authors:** Caitlin M. A. Simopoulos, Mitchell J. R. MacLeod, Solmaz Irani, Wilson W. L. Sung, Marc J. Champigny, Peter S. Summers, G. Brian Golding, Elizabeth A. Weretilnyk

**Affiliations:** 1 Department of Biology, McMaster University, 1280 Main Street West, Hamilton, Canada; 2 Current address: Department of Biochemistry, Microbiology and Immunology, University of Ottawa, 451 Smyth Road, Ottawa, Canada

**Keywords:** *Eutrema salsugineum*, *Thellungiella salsuginea*, Transcriptome, lncRNA, Extremophile, Drought stress

## Abstract

**Background:**

The severity and frequency of drought has increased around the globe, creating challenges in ensuring food security for a growing world population. As a consequence, improving water use efficiency by crops has become an important objective for crop improvement. Some wild crop relatives have adapted to extreme osmotic stresses and can provide valuable insights into traits and genetic signatures that can guide efforts to improve crop tolerance to water deficits. *Eutrema salsugineum*, a close relative of many cruciferous crops, is a halophytic plant and extremophyte model for abiotic stress research.

**Results:**

Using comparative transcriptomics, we show that two *E. salsugineum* ecotypes display significantly different transcriptional responses towards a two-stage drought treatment. Even before visibly wilting, water deficit led to the differential expression of almost 1,100 genes for an ecotype from the semi-arid, sub-arctic Yukon, Canada, but only 63 genes for an ecotype from the semi-tropical, monsoonal, Shandong, China. After recovery and a second drought treatment, about 5,000 differentially expressed genes were detected in Shandong plants versus 1,900 genes in Yukon plants. Only 13 genes displayed similar drought-responsive patterns for both ecotypes. We detected 1,007 long non-protein coding RNAs (lncRNAs), 8% were only expressed in stress-treated plants, a surprising outcome given the documented association between lncRNA expression and stress. Co-expression network analysis of the transcriptomes identified eight gene clusters where at least half of the genes in each cluster were differentially expressed. While many gene clusters were correlated to drought treatments, only a single cluster significantly correlated to drought exposure in both ecotypes.

**Conclusion:**

Extensive, ecotype-specific transcriptional reprogramming with drought was unexpected given that both ecotypes are adapted to saline habitats providing persistent exposure to osmotic stress. This ecotype-specific response would have escaped notice had we used a single exposure to water deficit. Finally, the apparent capacity to improve tolerance and growth after a drought episode represents an important adaptive trait for a plant that thrives under semi-arid Yukon conditions, and may be similarly advantageous for crop species experiencing stresses attributed to climate change.

## Background

A United Nations study group has recently reported that the number of undernourished people has increased around the globe since 2014, signaling a reversal in reductions for world hunger since 2005 [1]. A major contributing factor has been the impact of climate variation, including extreme climate-related events that have more than doubled in frequency during the past 30 years. Among climate-related disasters, drought has been especially impactful as it accounts for over 80% of all losses related to agriculture, and many nations most susceptible to drought have seen the greatest increase in undernourished people. A strong association between the prevalence of drought and increased food insecurity is not unexpected given that crop yield losses due to drought far exceed losses attributed to all other abiotic and biotic stressors [2]. Scientists predict that global climate change will likely exacerbate yield losses in the near future as drought episodes will continue to undergo increases in frequency and severity [3, 4]. Therefore, an improved understanding of how plants both respond to and recover from drought is vital to not only maintaining but also improving crop yields to meet a growing world population, one forecast to reach 9 billion by 2050 [5]. However, despite the growing need for crops that better manage water deficits, developing crops with improved drought tolerance has met with little success to date, in part because our basic knowledge of plant processes contributing to tolerance is poor and hence gene targets for crop improvement are, as yet, ill defined [6].

Plant responses to drought are complex and variable, but our understanding of this subject has advanced nonetheless, in part through the benefits accrued from using different experimental approaches. For example, using plants with documented physiological responses to an imposed stress allows for drawing correlative associations between the physiological and molecular responses to drought [7]. Meyer et al. [8] used a correlative approach with switchgrass to show that some genes only respond to drought-treatment exposures that extended beyond critical physiological thresholds (e.g. of water potential and photochemical quenching). Sequential drought treatments can also produce plants that display altered responses to subsequent exposures to water deficits [9].

The transcriptional response to repeated drought exposures can be distinct from the response to a single water deficit [10]. When *Arabidopsis thaliana* seedlings grown on media plates were exposed to repeated cycles of dehydration, the relative expression of several drought-responsive genes showed evidence of “training”, a phenomenon also referred to as “drought memory”. A genome-wide RNA-Seq approach helped resolve four distinct classes of drought memory genes in *Arabidopsis thaliana* that reflect their broad strategic roles in protecting plants from the deleterious aspects of drought [11].

In this report we describe the transcriptional responses of the extremophile crucifer *Eutrema salsugineum* (synonymous with *Thellungiella salsuginea*), to two water deficits separated by a brief recovery period. The geographic range where *Eutrema salsugineum* is found is broad and extends across the Asian and North American continents [12] and so, not surprisingly, across very different climatic conditions. In the semi-arid, sub-arctic Yukon, Canada, *Eutrema salsugineum* experiences periods with little precipitation in parts of its natural range [13]. In contrast, an accession originating in Shandong, China, is found in a temperate region that is subject to higher precipitation [14]. Importantly, both the Yukon and Shandong accessions are halophytes and are consequently equipped with a strong capacity for coping with high osmotic stress, and thrive when exposed to concentrations of NaCl exceeding 300 mM [15, 16]. Despite this unusually high tolerance to osmotic stress, MacLeod et al. [17] reported that the Yukon and Shandong *Eutrema salsugineum* accessions respond differently to a drought treatment that includes two periods of water deficit separated by a brief recovery period. Plants of the Yukon accession accumulate solutes in response to an initial water deficit and during a second drought treatment the plants retain water content longer and maintain leaf expansion. Conversely, plants of the Shandong accession show no obvious benefit from the initial drought exposure. These physiological responses are consistent with Yukon plants showing drought tolerance and Shandong plants displaying drought avoidance. Notably, the first drought exposure treatment did little to distinguish the drought-responsive phenotypes that characterize the two accessions.

An indication that the initial drought exposure elicits different responses at the molecular level between the Yukon and Shandong accessions was given by measures of gene expression for four genes classically found to be drought-responsive in many species namely *RAB18*, *RD29A*, *ERD1* and *RD22* [10, 18, 19]. Thus to extend the physiological research reported by MacLeod et al. [17], we undertook a comparative RNA-Seq study to provide a more complete understanding of how differently these two accessions respond to water deficits using plants exposed to the same progressive drought protocol. In this comparison we also evaluated the contribution of predicted long non-protein coding RNAs (lncRNAs), an interest prompted by their perceived and growing role as gene expression regulators during plant development and in response to stress, including water deficits [20–23]. Based on the RT-qPCR analysis of *RAB18*, *RD29A*, *ERD1* and *RD22* reported by MacLeod et al. [17], we hypothesized that the ecotypes would undergo different patterns of transcriptional re-programming during water deficits and that ecotype-specific lncRNAs may be implicated in their differential responses. In this work, we show that this prediction was borne out by comparative transcriptome analyses showing substantive differences in gene expression patterns of both protein-coding loci and lncRNAs that distinguish Yukon and Shandong *Eutrema salsugineum* plants with respect to their response to reduced water availability.

## Results

### RNA-Seq of *E. salsugineum* accessions following drought and recovery

We analysed leaf transcriptomes of Yukon and Shandong *Eutrema salsugineum* plants subjected to a progressive, two-stage drought treatment protocol that considers the fraction of transpirable soil water (FTSW) at harvest as described by MacLeod et al. (2015) [17]. Library names correspond to plants harvested as various FTSW percentages: WW1 (100% FTSW), severe drought at D1 (10% FTSW), following re-watering and recovering from drought at WW2 (100% FTSW), and a second severe drought at D2 (10% FTSW). This experiment included two different RNA-Seq library preparation protocols (See Methods). Table S1 shows that a comparable number of genes were detected in each of the cDNA libraries when considering both genotypes as well as the two different, albeit similar, library preparation methods. To confirm that sequencing timing did not interfere with gene expression detection, two previously prepared and sequenced cDNA libraries (SD2.2 and YD2.1) were resequenced (SD2.2b and YD2.1b). Using principal component analysis (PCA), few differences were observed between the two sequencing time points by way of library overlap visualized in a PCA biplot (Additional file 1: Fig. S1). However, as the pairs represented technical replicates, data from the resequenced libraries were not used in further bioinformatic analyses. Additionally, we assessed the capacity of our transcriptomic database to discern differentially expressed genes, particularly drought and/or accession-specific genes. To do so, we compared log_2_-fold change values derived by RNA-Seq data to expression data derived by an independent approach using RT-qPCR (Additional file 1: Fig. S2). We chose four genes for relative abundance determinations (*EsRAB18*, *EsRD22*, *EsRD29a* and *EsERD1*) as these four dehydrin-related genes were previously shown by RT-qPCR to distinguish the responses displayed by Shandong and Yukon ecotypes at various stages of the progressive drought protocol [17]. We found excellent agreement between RNA-Seq and RT-qPCR results for these four genes at the three stages tested (D1, WW2 and D2) relative to their levels of expression under control, WW1 conditions (Additional file 1: Fig. S1).

On average, approximately 17,400 genes were detected in each library with the lowest number of genes identified in the YWW2.1 library at 16,860 and the highest in SWW2.3 at 17,866 (Table S1) Using a minimum threshold for detection of 1 fragment per kilobase per million mapped reads (FPKM), we found read support for 20,841 genes, or 79% of the 26,531 genes comprising the predicted coding capacity of the Joint Genome Institute (JGI) *Eutrema salsugineum* v1.0 genome [24]. A number of genes (11%) were expressed only in Shandong (1268 genes) or Yukon *Eutrema salsugineum* leaves (1023 genes). Thus, for each accession, less than 5% of the total protein-encoding capacity of the genome was expressed in an accession-specific manner.

We did not restrict our analyses to reads mapping to annotated regions in the genome, but instead used a conservative approach to identify novel transcripts that are expressed but remain without annotation. In addition, we looked for expression of other transcripts previously described by Champigny et al. [25] and Yin et al. [26]. Of the 411 transcripts previously identified by Champigny et al. [25], 383 were expressed in at least one genotype and condition during the progressive drought (Table S2). An additional 1,608 previously unidentified transcripts, referred to as DLOCs in this work, were expressed at one point during the experiment, of which 24 were previously identified at identical genomic locations by Yin et al. [26]. Additionally, we detected the expression of 1,007 putative lncRNAs (Additional file 2), of which only 76 (7.5%) were present in the *Eutrema salsugineum* reference annotation.

### Identifying differentially expressed genes

**Fig. 1:**
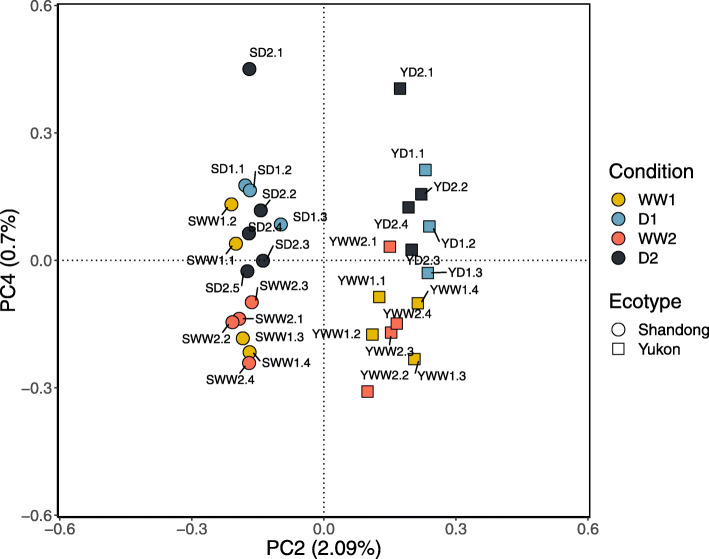
Principal component analysis biplot. PC2 vs PC3 biplot of transcript abundances of Yukon and Shandong *Eutrema salsugineum* plants undergoing stages of a progressive drought treatment protocol

PCA was used to explore sources of variance in transcript abundance among the 31 sequenced leaf cDNA libraries. PC1 accounted for 94.2% of the variance but did not distinguish the libraries on the bases of genotype or treatment, a feature that corresponds to gene expression levels common to all libraries, an observation also found by Champigny et al. [25] (Additional file 1: Fig. S3 and S4). In contrast to PC1, the variance explained by PC2, PC3 and PC4 (2.1%, 0.9%, 0.7%, respectively) accounted for far less of the total explained variance but offered more meaningful biological insights into genotype and treatment-specific differences between the transcriptomes (Fig. 1). By way of example, Fig. 1 is a biplot of PC2 and PC4 and it displays the variance due to ecotype, and to a lesser extent, variation due to treatment. Specifically, PC2 only explains 2.1% of the variance in the data but it clearly distinguishes the scores for Yukon transcriptomes from those of Shandong plants along the horizontal axis. For Yukon transcriptomes, PC4 discerned drought-treated from well-watered, including re-watered plants. The scores for cDNA libraries of drought-treated Yukon plants are positioned more positively along PC4 (YD1, YD2) whereas more negative scores are associated with plants that have either not experienced a water deficit (YWW1) or have been re-watered and allowed to recover following a drought treatment (YWW2). In contrast, the scores for Shandong libraries produced from well-watered plants (SWW1) cluster with plants experiencing drought (SD1, SD2) and re-watered plants (SWW2). Thus, PC4 appears to describe a source of variance that is related to water deficit for Yukon plants, with a far less clear distinction for the response to water deficits given by transcriptomes of Shandong plants.

**Fig. 2:**
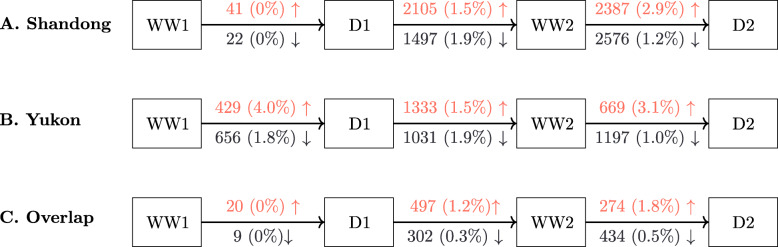
Number of shared and unique DEGs detected in each *E. salsugineum* ecotype. Number of DEGs detected in each *Eutrema salsugineum* ecotype and overlap between DEGs at each stage of the progressive drought treatment. The number of upregulated DEGs are described in coral above the transition arrow. The number of downregulated DEGs are given in blue below the transition arrow. Numbers in brackets refer to the percentage of DEGs predicted by CREMA as encoding lncRNAs

Analysis using **DESeq2** yielded 4,650 and 2,454 drought-responsive genes that were differentially expressed only in either Shandong or Yukon plants, respectively, while 1,599 differentially expressed genes (DEGs) were found in transcriptomes for both accessions (Additional file 3). Figure 2 provides an overview of differentially expressed gene DEG numbers identified in comparisons of the 31 transcriptomes over the course of the progressive drought protocol for each genotype separately (Fig. 2a,b) and as a summary of overlapping DEGs (Fig. 2c). The transition from a well-watered condition to D1 provides a striking impression. In Shandong plants, only 63 DEGs were identified as undergoing significant changes in expression after the first drought exposure, whereas 1,085 DEGs were detected in Yukon plants (Fig. 2a,b). A mere 29 DEGs were common between the two ecotypes. Figure 2 also provides the estimated contribution of DEGs predicted to be lncRNAs at each stage of the protocol for both natural accessions. Notably, none of the DEGs identified in Shandong plants at WW1 → D1 were predicted as lncRNAs, whereas 17 up- and 12 down-regulated lncRNAs (2.7% of 1,085 DEGs) detected in Yukon plants at WW1 → D1 were predicted as being lncRNAs. During the recovery from the initial drought (D1) to the re-watered and recovery stage (WW2), the two ecotypes again show different gene expression responses. Of the total DEGs identified in each genotype, over 82% and 77% were unique to Yukon and Shandong plants, respectively.

**Fig. 3:**
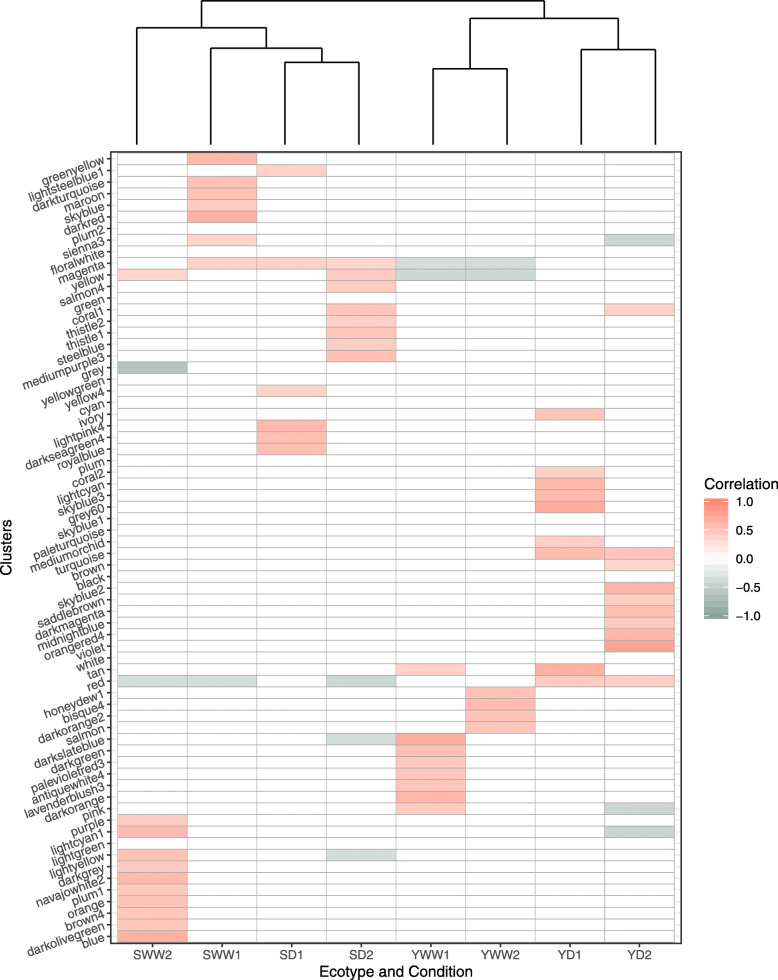
WGCNA cluster correlation heatmap. WCGNA cluster heatmap illustrating correlations of cluster eigengenes (Y axis) to ecotype and progressive drought conditions (X axis). Positive correlations are coloured in coral, negative correlations are coloured in slate and non-significant correlations are represented in white. Significance was defined as p < 0.05 after FDR adjustment. Samples were clustered using hierarchical clustering. A heatmap of correlations of select clusters is found in Fig. S5 (Additional file 1)

The overall impression is that Shandong and Yukon plants undergo different transcriptional reprogramming during both stages of the progressive drought protocol. Shandong and Yukon plants share as low as 2% of the DEGs identified at WW1 → D1 and up to 14% in D1 → WW2 plants. The overlap of DEGs predicted as lncRNAs is negligible as only 12 unique putative lncRNAs were identified among the DEGs of both *Eutrema salsugineum* genotypes (Fig. 2c; Additional file 3). We also tested whether any genes showed a similar pattern of drought-responsive expression during the progressive drought protocol. Figure 3 shows that only 13 DEGs displayed the same expression patterns, with eight showing increased transcript abundance following water deficit and decreased abundance under watered/re-watered conditions (Fig. 3a,b) while five DEGs showed the inverse response (Fig. 3c,d). For the eight DEGs undergoing increased transcript abundance with water deficit, the transcript levels in well-watered Yukon plants were typically already higher relative to those detected in well-watered Shandong plants, notwithstanding the drought-responsive increases found for both ecotypes (Fig. 3a,b).

### Correlating altered gene expression with biological responses to water deficit

We considered that the differential gene expression analysis summarized in Fig. 2 is limited to pairwise comparisons and hence would not identify groups of co-expressed genes that may contribute to insights into the unique drought responses of the Yukon and Shandong *Eutrema salsugineum* genotypes. As such, we performed a weighted gene co-expression network analysis (WGCNA) to cluster genes using estimated transcript abundances during the progressive drought treatment conditions. This “guilt-by-association” approach can help predict functionality of unannotated protein-coding or lncRNA-coding loci with information on the directionality (up- or down-regulated) of their possible roles during each treatment condition. For this analysis, expression estimates from all genes, as opposed to only DEGs, were used to allow for an unbiased, unsupervised clustering method. Eigengene values, summary statistics calculated using a dimensionality reduction method similar to PCA, were used to quantify the “average” gene expression values of each cluster. Using these cluster eigengene values, we correlated each gene cluster to drought treatment and ecotype (Additional file 4) and then selected clusters with 50% or more DEGs for gene ontology (GO) term enrichment analysis (Additional file 1: Table S3). One exception made in applying our selection of clusters for further analysis was the inclusion of the “red” cluster. The “red” cluster did not contain the requisite 50% DEGs, but it was enriched by expressed genes with a high correlation to the D1 and D2 treatments for Yukon but not Shandong plants and hence was expected to hold potentially novel, drought-responsive genes. A reduced list of highly significant biological processes in selected clusters was produced using REVIGO [27] and the results are summarized in Additional file 5.

**Fig. 4:**
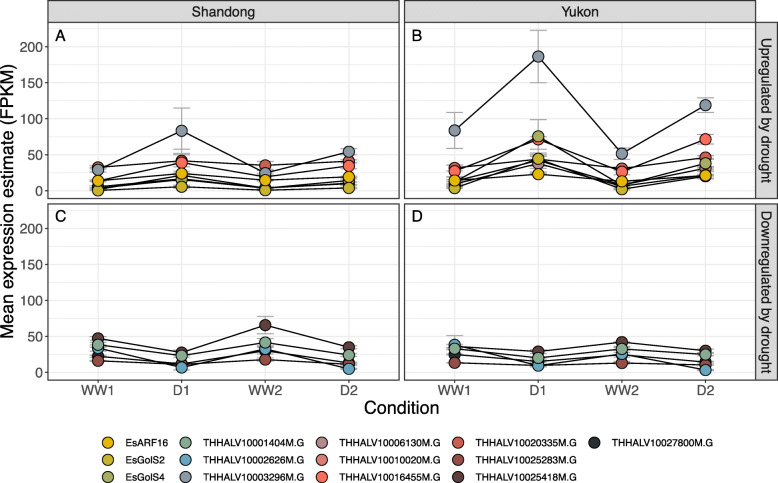
Average estimated FPKM values of DEGs following the same direction of fold change in both *Eutrema salsugineum* ecotypes. Average estimated FPKM values of DEGs identified by DESeq2 that follow the same direction of fold change in both *Eutrema salsugineum* ecotypes. Standard error of the expression values are represented by grey error bars n=3, 4, or 5 depending on condition

The heat map of cluster eigengene correlations to drought treatment (Fig. 4) shows correlations of ecotype and eigengenes grouping separately, suggestive of distinct responses to the progressive drought treatment by Shandong and Yukon plants. Moreover, the heat map also shows the correlated data for drought (D1, D2) and watered (WW1, WW2) treatments being grouped separately for Yukon plants whereas for Shandong plants the WGCNA results grouped both drought treatments together with the well-watered control (WW1) plants. Consistent with different physiological drought response strategies reported for the two ecotypes [17], only one cluster containing at least 50% DEGs, “coral1”, showed significant correlation to the same stage of drought treatment (D2) for both ecotypes (Fig. 4; Additional file 1: Fig. S5; Table S3). The “coral1” gene cluster is significantly enriched in GO terms relating to sulfur assimilation and sulfur utilization which infers a common connection between sulfur nutrition and a more prolonged exposure to drought stress (Additional file 5). Identifying only one ecotype-overlapping cluster suggests that groups of co-expressed genes are more highly correlated to a single ecotype and not shared by both ecotypes, an interpretation consistent with Shandong and Yukon plants expressing genes with different functions during drought. By way of example, genes of the “lightyellow” cluster are only correlated for the drought response of Shandong plants and interestingly, the clustered genes are negatively correlated with D2 but positively correlated with WW2 (Fig. 4; Additional file 1: Table S3). This associated set of correlated differences is particularly relevant given the DEGs summarized in Fig. 2a. Shandong plants did not significantly alter their gene expression during the initial drought exposure (D1) but a re-watering treatment following D1 and subsequent drought (D1 → WW2 → D2) triggered major transcriptional changes. The “lightyellow” cluster is composed of genes associated with metabolic processes, with the most significant GO terms associated with lipid biosynthetic processes, and ketone and carbohydrate metabolic processes (Fig. 4; Additional file 1: Table S3; Additional file 5). The directionality of the correlated transcriptional changes suggests that biosynthetic pathways promoted by re-watering were subsequently reversed by the second drought (D2).

For Yukon plants, co-expressed genes that positively correlated with both D1 and D2 are grouped in the “red” and “turquoise” clusters (Fig. 4; Additional file 1: Table S3). Containing 3,415 co-expressed transcripts, “turquoise” is the largest identified cluster yet contains only 2.1% genes predicted to be lncRNAs. While most genes in the “turquoise” cluster are annotated, 4.3% are novel or were previously identified as novel by Champigny et al. [25] or Yin et al. [26]. The “turquoise” cluster is enriched in genes with GO term annotation associated with water deprivation, peptide transport, and cellular lipid catabolic processes (Additional file 5). There were 1129 genes in the “red” cluster with the majority more highly expressed in Yukon relative to Shandong plants (Additional file 1: Fig. S6). No significantly enriched GO terms were identified in the red cluster, a reflection of a high contribution of genes with little to no annotation available in public repositories. Of the 1129 genes, 12.4% were predicted as lncRNAs and 35% were identified as “novel” genes.

The category “lightcyan1” offers a different type of response in being populated by genes negatively correlated to D2 in Yukon plants but also positively correlated to a re-watering (WW2) response in Shandong plants (Fig. 4; Additional file 1: Table S3). The “lightcyan1” cluster is comprised of genes associated with proteolysis and negative regulation of catalytic activity, offering an indication of related functions elicited during what should be a period of recovery for Shandong plants from water deficit as compared to the same response for Yukon plants under stress (Additional file 5). In contrast, both "purple" and "blue" clusters are only positively correlated to the re-watering (WW2) response for Shandong plants (Fig. 4; Additional file 1: Table S3). The “purple” cluster is highly enriched in genes associated with translation and DNA packaging and replication while genes in the “pink” cluster are related to cell cycle regulation, vitamin biosynthetic processes, nucleoside-related biosynthetic processes, and photosynthesis (Additional file 5). Thus the broad functionality of genes found in recovery-associated clusters of Shandong plants reflects the large transcriptional response of Shandong plants transitioning from D1 through WW2 (Fig. 2, Additional file 5). On the other hand, there is a notable lack of significant correlation between clusters of genes expressed by leaves of Yukon re-watered plants to any clusters primarily containing DEGs (Fig. 4; Additional file 1: Table S3) although Yukon plants, like Shandong plants, continued to grow during the entire progressive drought treatment [17].

## Discussion

MacLeod et al. [17] reported differences in the physiological responses of *Eutrema salsugineum* ecotypes exposed to an identical progressive drought protocol used in this RNASeq study. Yukon plants grown in controlled environment chambers were found to respond to an initial drought by a 46% reduction in stomatal conductance and 25% reduction in rosette water content relative to unstressed control plants, evidence of drought avoidance to conserve water [17]. Upon wilting, however, Yukon plants re-established turgor at significantly lower leaf solute potentials than the level for consistently well-watered Yukon plants which suggests osmotic adjustment and the development of drought tolerance. In contrast, while Shandong plants also showed signs of undergoing drought avoidance, leaf solute potentials in re-watered Shandong plants returned to pre-drought levels after re-watering, and unlike Yukon plants, evidence of drought tolerance was not found. Thus while the physiological responses of both ecotypes seemed similar during a first drought (D1), their distinct responses are more clearly seen during subsequent exposure to drought where Yukon plants take longer to lose turgor relative to Shandong plants. In this regard, the very different transcriptional responses that we observed, namely 1,100 DEGs detected in leaves of Yukon plants compared to only 63 in Shandong plants during a first drought (D1) was unexpected (Fig. 2a,b). Following recovery with re-watering (WW2) and subsequent exposure to a second drought (D2), 1,866 genes were differentially expressed in leaves of Yukon plants while Shandong plants now underwent a much larger transcriptional response with almost 5,000 genes showing differential expression. While there is overlap between transcriptional changes of both ecotypes, most of the DEGs were unique to each genotype (Fig. 2c).

The approach of using a progressive drought regime was valuable because, had we not exposed plants to a second water deficit, we would not have observed the large differences in overall drought-responsive gene expression between Shandong and Yukon plants. Similar progressive drought protocols have been used to study transcriptional reprogramming of alfalfa [28] and switchgrass [8]. However, these studies both show that fewer genes are differentially expressed during recovery from drought than during a severe water deficit. This is not consistent with our finding of a higher number of DEGs identified in both Yukon and Shandong plants that were re-watered (WW2) relative to plants undergoing an initial drought (D1) (Fig. 2a,b). This difference between the earlier work on alfalfa and switchgrass and our research with *Eutrema salsugineum* is especially evident in Shandong plants, where the 63 DEGs identified during D1 are followed by over 3600 DEGs in plants that were re-watered and allowed to recover.

One potential explanation that could explain the strong differential response by Shandong plants may relate to the extent by which these plants perceive the initial drought. Specifically, MacLeod et al. [17] found that physiological measurements of Shandong plants (including cut rosette water loss, static leaf water content, and specific leaf area) during D1 were not different from the same measurements of well-watered, control Shandong plants. This negligible physiological response suggests that Shandong plants were either not stressed or, more likely, the plants did not respond to the severity of water stress imposed by 10% FTSW and hence elicited few DEGs during the transition from SWW1 to SD1 (Fig. 2a). In that regard, the subtle, drought-responsive differences displayed by Shandong *Eutrema salsugineum* in our study are in agreement with the single water deficit treatment performed on this accession by Rosa et al. [29] leading the authors to conclude that this plant requires few adjustments in response to drought. However, the comparatively stronger transcriptional responses shown by Yukon plants during D1 and upon re-watering offer a contrasting interpretation and an example that the molecular adjustments for this species can be substantial (Fig. 2b). Moreover, the transcriptional changes seen for the Yukon accession during D1 are largely reversed during recovery, implying that what happens during drought is largely reversed during recovery. For example, 62% of the 429 genes up-regulated and 75% of the 656 down-regulated genes in Yukon plants identified during D1 showed altered expression in the opposite direction during re-watering. This observation led us to question why more genes show changed expression during D2 compared to D1 in Yukon plants (Fig. 2b). We originally predicted that the changed expression patterns of many genes may not return to pre-stress levels, and indeed 43% of the 429 genes up-regulated during D1 were also up-regulated during D2. However, when looking specifically at genes associated with drought for both ecotypes (Fig. 3) we see that genes induced during D1, although still differentially expressed in the second drought, show levels of expression that are lower in Yukon plants during D2. This behaviour is exemplified by two of the genes encoding dehydrins selected for RT-qPCR analysis, namely *EsRAB18* and *EsRD29A*, where drought-responsive changes in transcript abundance for Yukon plants are lower at D2 relative to D1 (Additional file 1: Fig. S2). Conceivably, whereas re-watering returns plants to the same water status achieved before drought, the transcriptional reprogramming during D1 has an enduring impact that may benefit Yukon plants during subsequent stress exposures.

MacLeod et al. [17] also reported that Yukon plants tolerate repeated drought exposure better than Shandong plants with benefits seen in solute accumulation and a longer time taken before turgor loss. However, the stress protective effect is not specific to drought. Exposure of Yukon plants to an initial drought treatment improves the freezing tolerance of Yukon plants from −19^∘^ C to −21^∘^ C with a shortened cold acclimation period [30, 31]. By not fully reverting to pre-stress levels, the constitutive expression of stress-responsive genes may enable the plant to retain a complement of gene products that serve as a “molecular buffer” for prolonged stress protection, products that may promote a greater coping capacity should the stress return. This further implies that Yukon plants, once stressed by exposure to water deficits, are no longer “naive” to stress and that their tolerance to other sources of adverse abiotic or biotic conditions can be improved. By way of contrast, the expression patterns for *EsRAB18*, *EsRD29A*, and *EsRD22* were very different in Shandong plants compared to Yukon plants (Additional file 1: Fig. S2). The ecotype-specific expression changes are particularly evident in the expression of *EsRAB18* and *EsRD29A* where their relative transcript levels remain high in Shandong re-watered plants (WW2), but are downregulated in Yukon plants experiencing the same re-watering treatment. This pattern of expression appears to be shared by a large number of drought-responsive genes given our finding of a large increase in gene expression changes in Shandong plants at WW2 and D2 (Fig. 2a). This different transcriptional response suggests that Shandong plants, unlike Yukon plants, may not be appropriately “primed” by the water deficit stress during D1 and, by consequence, are less able to cope with stress during the D2 treatment relative to Yukon plants.

We used WGCNA and the “guilt-by-association” approach to address the entire transcriptome in order to identify genes undergoing significant changes in expression during the progressive drought protocol for insight into their predicted functionality. By way of examples, cuticular waxes have been shown to be altered in a drought-responsive manner in a comparative study using Shandong and Yukon *Eutrema salsugineum* plants [32]. Xu et al. [32] reported that both *Eutrema salsugineum* ecotypes alter the composition and amount of cuticular waxes between non-stressed and drought-stress conditions with Yukon plants exhibiting a 4.6-fold increase in leaf wax content, although both ecotypes showed increases in the total amount of wax. MacLeod et al. [17] did not measure cuticular waxes in response to drought but rather focused on a variety of physiological changes including differences in accumulated solutes with drought exposure. Our WGCNA, however distinguished clusters of co-expressed genes relevant to the studies just described. The “lightyellow” cluster is negatively correlated to SD2 and positively correlated to SWW2 (Additional file 1: Table S3) and contained genes enriched in functions associated with ketone metabolic processes (Additional file 5). Indeed, the DEGs found in the “lightyellow” cluster were significantly increased in abundance in re-watered conditions and decreased in drought conditions in both Shandong and Yukon plants. While these transcriptional changes in the direction of expression of cuticular wax-related genes seems counter intuitive, studies exploring the regulation of wax biosynthesis in rice describe the gene DROUGHT HYPERSENSITIVE (*DHS*), encoding a RING-type E3 ligase, as a negative regulator of wax biosynthesis [33] and hence its overexpression reduces drought tolerance in transgenic rice lines. The gene DECREASE WAX BIOSYNTHESIS (*DEWAX*) is a transcriptional repressor of wax production with over-expression also reducing wax deposition [34]. Thus the enriched status of the “lightyellow” cluster by putative wax-related gene products may indicate that plants recovering from drought-stress are better positioned with respect to their capacity to alter cuticular wax composition and/or content, rather than a reflection of changes in the activity of wax-related biosynthetic processes themselves. Plants like Yukon *Eutrema salsugineum* that are adapted to dry environments are classically known to develop thick cuticular waxes but the regulatory mechanisms responsible remain a topic of considerable interest and are likely very complex as suggested in a recent review by Xue et al. [35].

Among the transcripts clustered by WGCNA were lncRNAs. lncRNAs are proposed to function as gene expression regulators, particularly in organisms experiencing stress [36]. In this study, the two *Eutrema salsugineum* ecotypes display different transcriptional responses to water deficits. Hence, we predicted that there should be some differences in lncRNAs expression in the plants undergoing the progressive drought treatment. Unexpectedly, we found an almost complete lack of overlap in the drought-associated lncRNAs expressed in Yukon and Shandong plants (Fig. 2c). This finding of negligible overlap among lncRNAs is perhaps not surprising given their fast evolution [37] and the extreme conditions that have likely led to the local adaptation of *Eutrema salsugineum* ecotypes to different natural environments. The known association between stress and lncRNAs expression also led us to predict that more lncRNAs would be expressed by plants subjected to water deficits, particularly after two drought exposures (D1 and D2). Surprisingly, this was not the case. Instead, we detected 1,007 lncRNAs from transcriptomes of both ecotypes, with 760 and 834 found in Yukon and Shandong plants, respectively. While we might expect most lncRNAs to appear after experiencing a water deficit, 627 and 684 lncRNAs were already expressed in well-watered, or “control”, Yukon and Shandong plants, respectively. Thus over 60% of the lncRNAs that we detected in both *Eutrema salsugineum* ecotypes were also expressed in plants that we have no reason to believe were stressed.

lncRNAs are notoriously difficult to identify with certainty and, not surprisingly, are generally poorly annotated in existing public plant reference genomes [38]. For this study we used an accurate ensemble machine learning program (CREMA [39]) that used validated lncRNAs for training. It is possible that we did not detect the complete set of lncRNAs and so underestimate their contribution to the libraries from stressed plants. However, it is reasonable to expect the same underestimate to apply to the libraries from unstressed plants if the lncRNAs identification means was a concern. A more likely source of uncertainty is discussed by Cui and Xiong [40] who raise the potential for inadequately spliced gene variants to be produced under stress conditions and, as such, it is possible that products lacking open reading frames could be counted as lncRNAs. Our choice of a 1 FPKM expression threshold led us to remove 257 and 235 lncRNAs in well-watered (WW1) Yukon and Shandong plant libraries, respectively, and so are not included in the 627 and 684 total putative lncRNAs reported above (Additional file 2). D1, WW2 and D2 libraries had much higher failure rates, in that many more putative lncRNAs showed very low or excessive variability in expression levels, and again were not included in FPKM counts. In addition to the 77 predicted lncRNAs that we found in Shandong libraries, we excluded 303 products, or roughly 4-fold as many predicted lncRNAs did not meet our threshold. The failure rate was almost 2-fold higher for the Yukon libraries (522 failed the FPKM threshold and only 58 passed).

Expression levels of *A. thaliana* lncRNAs were also found to be significantly lower than coding RNAs and their expression was found subject to specific stress and/or developmental conditions [41]. Low, condition-specific expression and improper pre-mRNA processing in transcriptomes of plants that have experienced stress are considerations that raise general concerns about determining the full and reliable impact of adverse conditions on lncRNAs expression. However, whether we include or exclude all of the lncRNAs that failed our expression level 1 FPKM threshold, we detected higher lncRNAs expression in *Eutrema salsugineum* plants that were not deliberately stressed relative to those that were subjected to the progressive drought. Constitutive expression of protein-coding genes in *Eutrema salsugineum* that are found to be stress-responsive in many plant species is a well documented attribute of *Eutrema salsugineum* [14, 30, 31, 42] and our results suggest that constitutive expression of lncRNAs may also hold true in spite of the fact that these two extremophyte ecotypes are sufficiently diverged that overlap among the expressed lncRNAs is negligible (Fig. 2c). This proposal of constitutive expression has some empirical support in a study of the Yukon *Eutrema salsugineum* response to low phosphate relative to similarly treated *A. thaliana*. Using absolute quantitative RT-qPCR, Velasco et al. [42] reported that the expression of the gene encoding the lncRNAs Induced by Phosphate Starvation 2 (*IPS2*) was detected in leaves of phosphate-fertilized plants at a level roughly equal to the “induced state” of *IPS2* expression for *A. thaliana*. Moreover, while *IPS2* transcript abundance was elevated under low phosphate conditions for both species, in phosphate-fertilized *A. thaliana* transcripts corresponding to *IPS2* were not detected. It will be interesting to determine in future studies of *Eutrema salsugineum* whether other stress or development-responsive lncRNAs show the same pattern of constitutive expression with comparatively modest, condition-dependent adjustment in their abundance.

With respect to the clustering produced by WGCNA, we particularly focused our analysis on genes associated with the “turquoise” cluster, a group positively correlated to D1 and D2 drought treatments in Yukon plants. The “turquoise” cluster was functionally enriched in genes with GO terms associated with plant response to water deprivation, including responses to abscisic acid (ABA) (Additional file 5). The “turquoise” cluster contains 36 differentially expressed putative lncRNAs, 30 of which are up-regulated during both drought treatments. Eight of the drought-induced lncRNAs are only differentially expressed in Yukon plants whereas 18 are specific to Shandong plants, further indication that both ecotypes deploy distinct lncRNAs in response to the same stress treatment protocol. The “turquoise” cluster was enriched in GO terms with functions similar to a previously identified lncRNAs, drought induced lncRNA (*AtDRIR*), thus far only detected in *A. thaliana* [23]. We did not find evidence of genes in this cluster with sequence homology to *AtDRIR* indicating the gene products we describe are most likely previously unidentified water deficit stress-associated lncRNAs transcripts.

Interestingly, we found only 13 drought-responsive genes that display similar expression patterns in both Shandong and Yukon plants. Of the eight genes that display a positive response to drought (Fig. 3a,b), all but one, Thhalv10020335m.g, are found in the “turquoise” cluster that is enriched in drought-related genes. We explored the functions of the overlapping genes as we hypothesized that these products may be part of a conserved drought response for *Eutrema salsugineum* and likely other plants. Thhalv10024122m.g, is homologous to the *A. thaliana* gene AT2G38800.1 and encodes a plant calmodulin-binding protein that has previously been characterized by Lovell et al. [43] as a quantitative trait locus (QTL) associated with drought in *A. thaliana*. Thhalv10003296m.g (AT5G43150) is a predicted mitochondrial protein with no known function, however, this gene is expressed under a variety of abiotic stresses in both *A. thaliana* and *Oryza sativa*, consistent with a role in a conserved stress response for plants [44]. Using a combined expression ranking and co-expression analysis, Ransbotyn et al. [45] also identified AT3G57540 (Thhalv10006130m.g) to be stress responsive, and found its expression to cluster with other ABA-responsive genes, a finding similar to this work. Thhalv10023585m.g (AT1G60470) and Thhalv10024122m.g (AT5G43150) are both annotated as encoding galactinol synthases, enzymes involved in the biosynthesis of raffinose family oligosaccharides, known osmoprotectants in plants [46] and hence likely playing a similar role in osmoprotection for *Eutrema salsugineum* experiencing drought. Galactinol and raffinose accumulate during stress treatments in leaves of *Eutrema salsugineum* and these metabolites are detected in *Eutrema salsugineum* plants collected at a highly saline Yukon field site [13]. Rasheed et al. [47] identified AT1G34060 (Thhalv10010020), a tryptophan aminotransferase, to be upregulated during drought, as well as other auxin-related genes, similar to Thhalv10024601 (AT4G30080), an auxin response factor. Thus the comparatively small group of drought-induced genes shared by both ecotypes are well-known to be associated with osmotic stress. Of additional interest for this group of drought-responsive genes is the differences in their expression levels between the two ecotypes with the comparatively muted transcriptional changes detected for Shandong plants relative to Yukon plants with drought stress (Fig. 3).

## Conclusion

Although Yukon and Shandong *Eutrema salsugineum* plants are both halophytes, several studies, including this work, show that they do not respond similarly to stress. At the physiological level, these *Eutrema salsugineum* ecotypes modulate their photosynthetic responses to light and temperature differently [31] and they respond to water deficits by differential alterations in wax composition and water use [17, 32]. As discussed earlier, there is evidence that Yukon *Eutrema salsugineum* benefits from an improved, or “primed”, physiological response upon exposure to drought or freezing temperatures following exposure to a water deficit [17, 30]. We show evidence for global transcriptome reprogramming during a second drought treatment (D2) in Yukon plants that is remarkably different and comparatively more constrained relative to the response shown by Shandong plants, the latter being an ecotype that does not display drought-mediated priming [17] and shows more modest adjustments with drying soil relative to drought-sensitive Arabidopsis [48]. Conceivably, a capacity for up-regulating a constitutive state of tolerance to abiotic stress by inducing a “stress memory” would be advantageous to the Yukon *Eutrema salsugineum* ecotype in its natural habitat where soil water is typically scarce, rainfall is unpredictable, and daily minimum temperatures below 10^∘^C are the norm [13].

Presumably there are costs associated with sustaining a “primed” state of tolerance, particularly under the extreme Yukon field conditions to which this plant has adapted. However, as yet there are no studies addressing factors affecting fitness of the Yukon ecotype, particularly under field conditions. Moreover, given the extreme nature of the Yukon soil and climate, one might expect the unprimed condition to be an anomaly found under the controlled, highly unnatural conditions of the laboratory and perhaps never seen in the continually changing Yukon habitat. Little is known about how priming is induced in plants and yet the enhanced level of stress tolerance it would confer is viewed as an important trait to improve the stress tolerance of crops to the adverse conditions of climate change [49]. In our study, among the differentially expressed genes that we detected, most were ecotype-specific and many genes were only expressed following drought exposure. Among this group of Yukon ecotype-specific, drought-responsive transcripts were protein-coding and putative lncRNAs, the latter a class of cryptic products known by reputation to regulate critical developmental and stress-related processes in diverse organisms. Future research comparing transcriptomes of field collected and cabinet-grown *Eutrema salsugineum* plants subjected to a multi-stage drought protocol could discern the genetic and physiological mechanisms that underlie priming, with potential insights from an extremophile plant informing strategies for improving the tolerance of field crops to drought.

## Methods

### Plant growth conditions and drought simulation assay

Plant growth conditions and relevant physiological measurements were previously reported in MacLeod et al. [17]. Briefly, Shandong and Yukon *Eutrema salsugineum* (Pall.) Al-Shehbaz & Warwick plants were grown in a controlled environment growth chamber and subjected to a drought simulation assay consisting of two periods of water deficit separated by a two-day recovery period [17]. The progress of the drought treatment was monitored gravimetrically and the FTSW was determined. FTSW was maintained at approximately 100% over the course of the entire experiment for well-watered, control plants (WW1). Plants undergoing drought treatment were water-deprived until FTSW reached 10% (D1). Plants were allowed to wilt (0% FTSW under our growth conditions), then were re-watered and given 48 h to recover with soil kept at about 100% soil water holding capacity (WW2). After recovery, water was again withheld from plants to begin the second drought treatment and FTSW again reached 10% (D2). The use of FTSW measurements as a guide for harvesting plants ensured that plants from both accessions were exposed to a comparable severity of soil water deficit.

### Selection of leaves for transcriptome profiling

Only fully-expanded rosette leaves were harvested from both Yukon and Shandong plants. Leaf samples used for RNA extraction were collected between 8 and 10 h into the day cycle under our cabinet conditions. Once harvested, the leaves were flash-frozen in liquid N and then transferred to a freezer for long term storage at −80^∘^ C. Leaves were harvested at the following stages: control plants watered daily to match water lost by transpiration (WW1) and those undergoing D1, D2, and WW2 treatments. One control plant was harvested when the drought-treated plants reached D1 and four plants at the WW2 (recovery stage) of the drought protocol. The difference in age between these control plants was 5 days.

### cDNA library construction, transcriptome assembly, and RT-qPCR

For library preparation, total RNA was extracted from frozen leaves using a hot borate method [50] modified as described in Champigny et al. [25]. RNA quantity and integrity was assessed using RNA Nano 6000 chips on a Bioanalyzer 2100 instrument. Two mRNA purification procedures were performed with slightly modified protocols because replicate samples were processed and sequenced on two different dates: A. Three successive on-column purifications using the Genelute mRNA miniprep kit (Cat. No. MRN10, Sigma) or B. NEBNext Poly(A) mRNA Magnetic Isolation Module (E7490). Both mRNA purification protocols were followed by the NEBNext Ultra II Directional RNA Library Prep Kit for Illumina (E7760). In order to ensure reproducibility of data between the two sequencing runs, we randomly selected two cDNA libraries to sequence on both dates. As described in Results, only data generated during the first sequencing run of the two technical replicate samples were used for statistical analyses.

In total, RNA-Seq data from 31 cDNA libraries were compared with a minimum of three cDNA libraries for each ecotype and treatment stage. The libraries are referred to by accession (Y or S), drought-treatment stage (WW1, D1, WW2, or D2), and plant number (1, 2, 3, 4, or 5; See Fig. 1). The technical replicates sequenced twice correspond to cDNA libraries of plants YD2.1 and SD2.2 (Figure S1b). cDNA libraries were prepared using the NEBNext multiplex cDNA synthesis kit for Illumina using random hexamers (Cat. No. E7335, New England Biolabs, Ipswich, MA). The cleanup of fragmented RNA was performed with Agencourt AMPure XP Beads (Cat. No. A63987, Beckman Coulter, Mississauga, ON) following the manufacturer’s protocol. A combination of two high-output, paired-end sequencing runs of 100 or 150 bp length were performed using the Illumina Hi-Seq 1500 platform. Quality control, amplification, and sequencing of the cDNA libraries were conducted at the sequencing facility of the Farncombe Family Digestive Health Research Institute (McMaster University, ON, Canada).

Following sequencing, the reads obtained were trimmed of Illumina adaptor sequences and low quality reads with default settings of Trimmomatic v0.34 [51]. Only reads ≥36 bp after trimming were mapped to the JGI *Eutrema salsugineum* genome assembly downloaded from Phytozome v12.1 [24, 52] using STAR v2.5.2b [53]. We used StringTie 1.3.4d [54] for transcript assembly on each individual RNA-Seq library to identify transcripts missing from the *Eutrema salsugineum* reference annotation. Library-specific transcripts called by StringTie were merged with each other and the reference annotation using StringTie **–merge** default settings.

Assembled transcripts that were not found in the *Eutrema salsugineum* reference annotation, or were not previously identified by Champigny et al. [25], were considered novel transcripts and have the locus identifier-prefix “DLOC”. The merged GTF annotation file that included all novel transcripts, those identified by Champigny et al. [25], and those found in the reference annotation was created and used for consequent transcript abundance estimates and is available in Additional file 6. The merged GTF file was compared to the reference annotation using **gffcompare** and only those transcripts classified as “unknown” and “intergenic” were retained for further analysis to reduce errors caused by mapping, assembling, or sequencing of unprocessed transcripts.

Total template cDNA was prepared from the same RNA samples used for RNA-Seq analysis. cDNA was synthesized from 1 µg of total RNA in a 20 µL reaction volume using iScript gDNA Clear cDNA synthesis kit (Bio-Rad, USA). Gene-specific primers used were as reported by MacLeod et al. [17] including those for reference genes *EF1a* (Thhalv10013526), *UBQ* (Thhalv10006290) and *YL8S* (Thhalv10014963m). Real-time quantitative PCR (RT-qPCR) reactions were performed using SYBR Green Supermix (Bio-Rad, USA) according to the manufacturer’s instructions.

### Determination of transcript abundance

Gene expression estimates for transcripts that met our classification criteria (i.e. in reference annotation, “unknown” or “intergenic”) were calculated using RSEM v1.2.31 [55] and an internal call for mapping to the transcriptome using RNA-Seq aligner STAR v2.5.2 [53] to accommodate the ambiguity of multi-mapping reads. Gene level transcript abundance is reported as the number FPKM, a determination accounting for both mRNA length and library size [56]. FPKM estimates for each gene were calculated using expected counts and the median length of each transcript considering all RNA-Seq libraries.

### lncRNA prediction

Single nucleotide polymorphisms (SNPs) were also called separately for both *Eutrema salsugineum* ecotypes following GATK’s best practices [57] for RNA-Seq data (Accessed September 1, 2018). Reads were mapped to the *Eutrema salsugineum* genome, rather than transcriptome, with STAR v.2.5.2b [53] using splice site junctions identified by the first read mapping step as suggested by GATK’s best practices. SNPs were called individually for each library and were merged into a genotype-specific single variant calling file for downstream analyses. SNPs were filtered using GATK’s **VariantFiltration** software to flag: clusters of three or more SNPs in 35 base pair windows, QualByDepth (QD) <2 and FisherStrand (FS) >30. Using a custom Python script, only homozygous SNPs that were not flagged and found in the majority of each genotype’s cDNA libraries were retained. VCFtools [58] was used to create new genome consensus files for each genotype containing the consensus filtered SNPs. Transcript sequences for each ecotype were extracted using the individual genotype genome files and merged annotation files. Each transcript was then input into CREMA (https://github.com/gbgolding/crema) [39] for lncRNAs prediction. Utilizing CREMA’s numerical scoring system for lncRNAs prediction, only those transcripts with a prediction score > 0.5 were considered putative lncRNAs.

### Multivariate analysis

Statistical analyses on gene expression data was performed using R v3.5.1 [59] using FPKM values shifted by a constant of 1 to allow the data to be log_2_ transformed. Normalization was used to account for the disparity in transcript abundance for genes with very low or very high expression. PCA was performed on the covariance matrix for all genes detected across the 31 RNA-Seq libraries (technical replicates removed) of both *Eutrema salsugineum* ecotypes subjected to the progressive drought treatment in order to explore variation within and between the transcriptomes with regards to gene expression estimates. Log_2_ transformed FPKM values for transcripts associated with 28,712 genes were treated as variables while each of the 31 cDNA libraries were treated as observations.

### Detection of differentially expression genes

DEGs were called using the **DESeq2** Bioconductor package [60] using a false discovery rate (FDR) threshold of 0.05 [25]. To control for a potential batch effect due to differences in library preparation protocols, library preparation type was added to the **DESeq2** regression formula. In addition, a threshold was set for differentially expressed genes to reduce predictive error that may arise with biological variance. In a differential expression test between Condition A vs Condition B, all genes identified as “upregulated” must have gene expression estimates > 1 FPKM in Condition A. Similarly, all genes identified as “downregulated” must have gene expression estimates > 1 FPKM in Condition B. DEGs were identified in all biologically relevant drought progression transitions (i.e WW1 vs D1, D1 vs WW2, WW2 vs D2). The script used for data analysis is available at https://github.com/caitsimop/eutrema_drought_transcriptomics.

### Weighted gene co-expression network analysis and gO term enrichment

A gene co-expression network was inferred from untransformed FPKM values of all expressed transcripts using the **WGCNA** R package [61]. A “signed hybrid” network was constructed in a blockwise manner using a maximum block size of 10,000 genes, a soft threshold power of 9, minimum module size of 30, and a merge cut height value of 0.25. Gene expression clusters were summarized using an eigengene value equal to the first principal component of gene expression values contained in each cluster. Cluster eigengene values were correlated to each ecotype’s progessive drought treatment status in order to identify genes associated with an ecotype and/or drought treatment. Correlation of treatments was also clustered using hierarchical clustering. We chose to focus our investigation on nine clusters: eight clusters composed of over 50% DEGs and one cluster with a high correlation to drought stress (Additional file 1: Table S3).

The DEG-containing clusters were used to identify significantly enriched GO terms based on custom GO term annotation. Because homology with *A. thaliana* genes was used for GO term assignment, we used a reciprocal best BLAST hit approach to annotate novel transcripts identified in our cDNA libraries with *A. thaliana* loci. The most recent *A. thaliana* GO terms were downloaded from TAIR on November 12, 2018 (https://www.arabidopsis.org/download/index-auto.jsp?dir=/download_files/GO_and_PO_Annotations). *Eutrema salsugineum* genes were annotated with *A. thaliana* GO terms using *A. thaliana* loci available from Phytozome v12.1.5, annotation provided by Champigny et al. [25] and the annotation of novel transcripts by reciprocal best BLAST hit. GO term enrichment of each of the eight clusters was called using the **topGO** R package [62] using the Benjamini and Hochberg [63] FDR set at a 0.05 significance threshold. Redundancy of enriched GO terms was reduced using the Revigo webserver [27], using the *A. thaliana* GO term database size and an allowed SlimRel maximum measure of 0.4. FDR adjusted p-values of the enriched GO terms were also used in the GO term summary process. The script used for data analysis is available at https://github.com/caitsimop/eutrema_drought_transcriptomics.

## Supplementary information

**Additional file 1** Supplemental figures and tables.

**Additional file 2** List of predicted lncRNAs.

**Additional file 3** Significant DEGs identified at all biologically relevant drought progression stages.

**Additional file 4** DEG composition of all clusters.

**Additional file 5** GO enrichment of selected clusters.

**Additional file 6** New *Eutrema salsugineum* genome annotation containing XLOCs identified by Champigny et al. [25] and novel DLOCs identified in this study. File is set up in same column order as.gtf format.

## Data Availability

Raw FASTQ RNA sequencing data is available in the SRA in BioProject Accession PRJNA494564 and SRA numbers SRR7962298, SRR10174220, SRR10174221, SRR10174223, SRR10174224, SRR10174225, SRR10174234, SRR10174226, SRR10174227, SRR10174228, SRR10174233, SRR10174229, SRR10174230, SRR10174232, SRR10174235, SRR10174236, SRR10174237, SRR10174238, SRR10174243, SRR10174239, SRR10174242, SRR10174240, SRR10174241, SRR10174214, SRR10174215, SRR10174216, SRR10174217, SRR10174218, SRR10174219, SRR10174222, SRR10174231, SRR10174244, and SRR10174245. The code used for statistical analyses and all required data files are found at https://github.com/caitsimop/eutrema_drought_transcriptomics.

## Abbreviations

ABA: Abscisic acid
CREMA: Classifying RNAs by ensemble machine learning algorithm
D1: First drought treatment
D2: Second drought treatment
DEG: Differentially expressed gene
*DEWAX*: DECREASE WAX BIOSYNTHESIS
*DHS*: DROUGHT HYPERSENSITIVE
*DRIR*: Drought induced lncRNA identified in *A. thaliana*
FDR: False discovery rate
FPKM: Fragment per kilobase per million mapped reads
FTSW: Fraction of transpirable soil water
GO: Gene ontology
GTF: Gene transfer format
*IPS2*: Induced by phosphate 2
JGI: Joint Genome Institute
lncRNA: Long non-protein coding RNA
PC: Principal component
PCA: Principal component analysis
QTL: Quantitative trait locus
S: Shandong ecotype
SNP: Single nucleotide polymorphism
WGCNA: Weighted gene co-expression network analysis
WW1: Well watered control treatment
WW2: Re-watering treatment
Y: Yukon ecotype
