# Supplementary material for: Coding and long non-coding RNAs provide evidence of distinct transcriptional reprogramming for two ecotypes of the extremophile plant Eutrema salsugineum undergoing water deficit stress
Source: BMC Genomics. 2020 Jun 8;21:396. doi: 10.1186/s12864-020-06793-7 (PMC7278158; doi:10.1186/s12864-020-06793-7)
Supplement: Supplementary file 1 — Additional file 1 Supplemental figures and tables. [file 12864_2020_6793_MOESM1_ESM.pdf]

Table S1: Number of detected loci by RNA-Seq in all 31 RNA-Seq libraries at a threshold of > 1 FPKM

	All loci		Putative lncRNA	
Library	Yukon	Shandong	Yukon	Shandong
WW1.1[a]	17268	17749	446	498
WW1.2[a]	17303	17440	462	520
WW1.3[b]	17106	17595	442	480
WW1.4[b]	17320	17423	475	489
D1.1[a]	17608	17512	509	489
D1.2[a]	17587	17479	522	449
D1.3[b]	17170	17532	459	534
WW2.1[a]	16860	17118	445	484
WW2.2[a]	17545	17424	514	510
WW2.3[b]	17217	17866	462	548
WW2.4[b]	17037	17758	437	524
D2.1[a]	17484	17589	517	531
D2.2[a]	17493	17516	507	534
D2.3[b]	17367	17277	477	520
D2.4[b]	17312	17075	470	483
D2.5[b]	NA	17357	NA	509
Mean	17311	17481	486	506

[a] Library preparation protocol A
[b] Library preparation protocol B

Table S2: Number of detected unannotated genes in all 31 RNA-Seq libraries at a threshold > 1 fragment per kilobase per million mapped reads (FPKM)

	Champigny et al. [2013]		Yin et al. [2018]		DLOC	
Library	Yukon	Shandong	Yukon	Shandong	Yukon	Shandong
WW1.1	308	287	15	23	650	722
WW1.2	316	281	15	23	668	763
WW1.3	298	274	13	23	643	697
WW1.4	312	272	14	22	695	711
D1.1	316	286	14	23	767	717
D1.2	322	292	14	22	798	639
D1.3	302	280	14	21	671	794
WW2.1	307	287	14	22	654	675
WW2.2	310	285	14	22	760	731
WW2.3	307	281	14	20	670	793
WW2.4	300	278	14	20	637	771
D2.1	316	296	13	20	776	756
D2.2	319	301	13	22	757	776
D2.3	307	281	14	22	729	764
D2.4	305	272	13	22	706	687
D2.5	NA	274	NA	22	NA	727
Mean	310	283	14	22	705	724

Counts include 24 loci annotated by Yin et al. [2018], 383 loci previously reported by Champigny et al. [2013] and 1608 novel loci (DLOCs) identified by this study.

Table S3: Correlation of select cluster eigengenes to genotype and drought treatment

Cluster	Number of genes			Correlation to Condition							
	DEGs		Total	SD1	SD2	SWW1	SWW2	YD1	YD2	YWW1	YWW2
lightcyan1	121	(89%)	136	ns	ns	ns	0.56	ns	-0.45	ns	ns
lightyellow	184	(76%)	241	ns	-0.36	ns	0.53	ns	ns	ns	ns
purple	393	(75%)	525	ns	ns	ns	0.43	ns	ns	ns	ns
pink	444	(66%)	677	ns	ns	ns	ns	ns	-0.43	0.43	ns
blue	2008	(61%)	3311	ns	ns	ns	0.66	ns	ns	ns	ns
darkslateblue	63	(55%)	115	ns	-0.36	ns	ns	ns	ns	0.70	ns
turquoise	1756	(51%)	3415	ns	ns	ns	ns	0.57	0.51	ns	ns
coral1	42	(51%)	83	ns	0.48	ns	ns	ns	0.40	ns	ns

Clusters for GO term enrichment were chosen if at least 50% of genes in each cluster were identified as a differentially expressed gene (DEG) at one progressive drought condition progression. Only significant correlations are displayed (p<0.05 after FDR adjustment). NS indicates that the correlation was not significant.

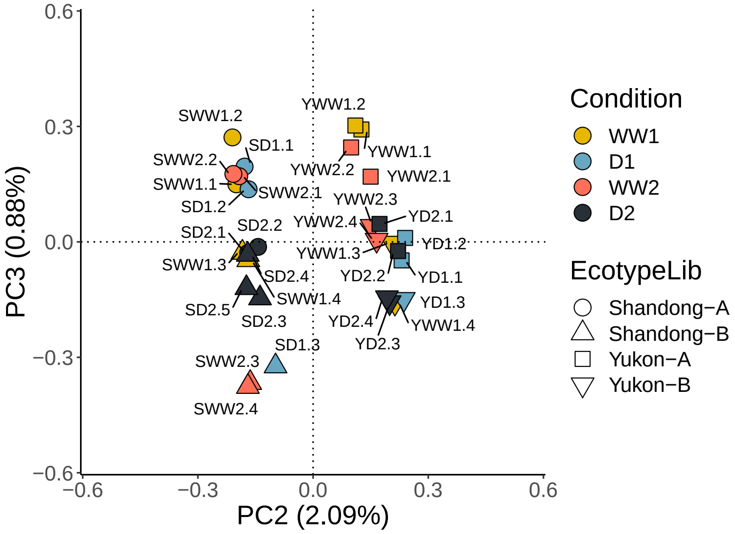

(a)

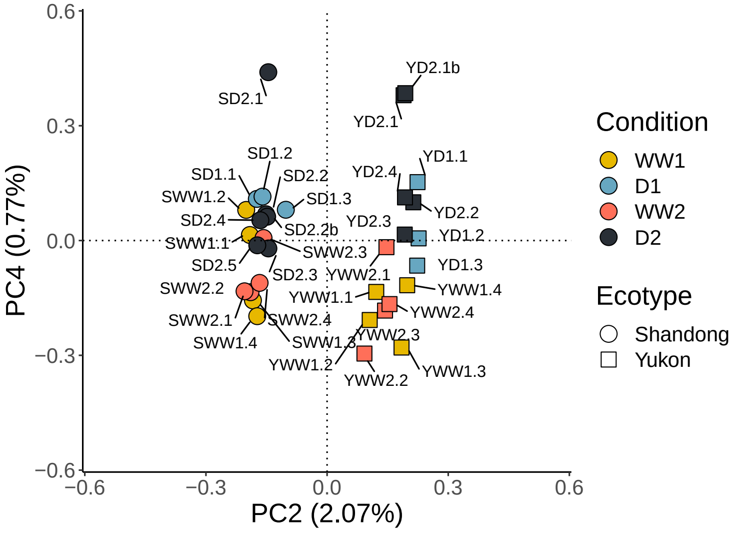

(b)

Figure S1: PCA biplots used to identify possible batch effect caused by different cDNA library preparation protocols. a. PC2 and PC3 biplot that shows clustering of libraries sequenced at different times and prepared using different library preparation protocols. cDNA libraries prepared by protocol A are shown in circles (Shandong) and squares (Yukon). cDNA libraries prepared by protocol B are shown in upwards (Shandong) and downwards (Yukon) facing triangles. Library preparation A can be found clustering positively on PC3, while library preparation B is negatively scoring on PC3. The clustering was used for batch effect detection. Batch effect was considered in the DEG analysis using DESeq2. b. PC2 and PC4 biplot shows overlapping technical replicates of resequenced cDNA libraries (YD2.1 and YD2.1b, and SD2.2 and SD2.2b) suggesting that it is library preparation methods, not sequencing technologies, that are causing a putative batch effect.

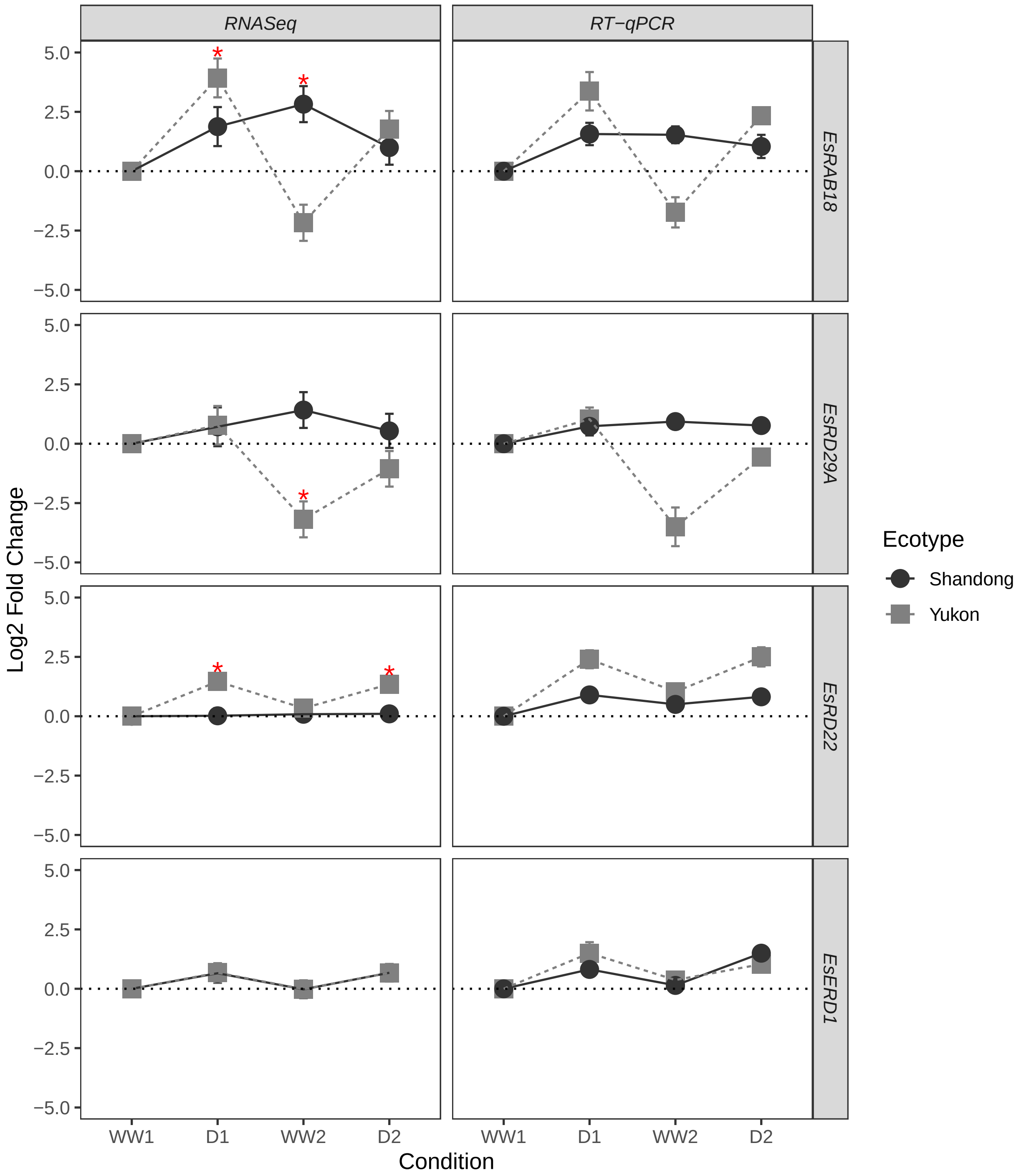

Figure S2: Log_2 fold change of the dehydrins described by MacLeod et al. [2015]. All $\log_2$ estimates are relative to WW1, or control, conditions for each ecotype. Significant fold changes are described by a red asterisk (*). Error bars represent the standard error of the $\log_2$ fold change. Log_2 fold change results of RNASeq data from all 31 libraries were identified using DESeq2 and an FDR adjust p-value threshold of 0.05. Log_2 fold change results of RT qPCR data of three biological replicates from each condition were identified using a t-test and an FDR adjusted p-value threshold of 0.05

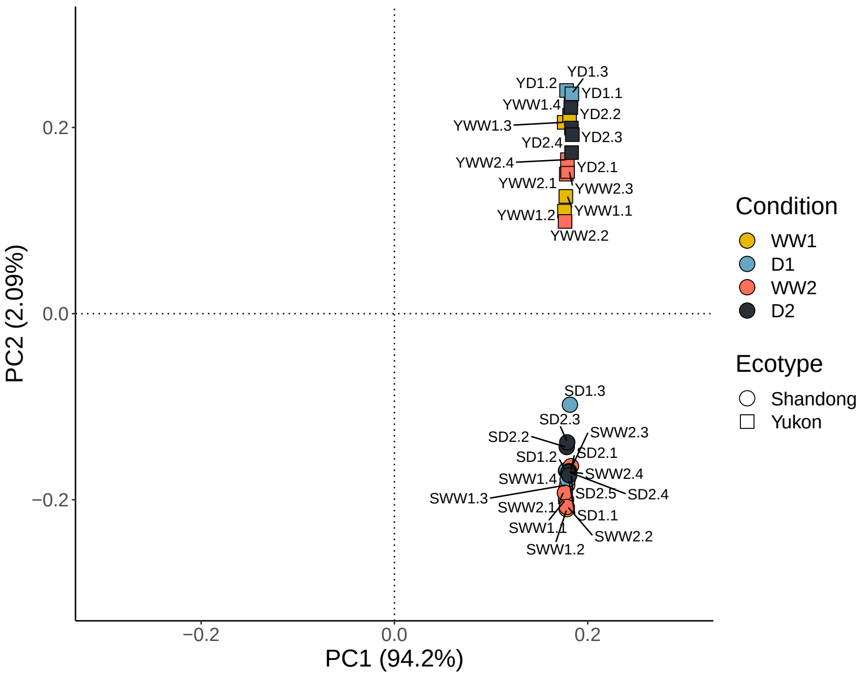

Figure S3: A PCA biplot of PC1 vs PC2 depicting RNASeq library scores. Shandong and Yukon libraries are represented by circles and squares respectively.

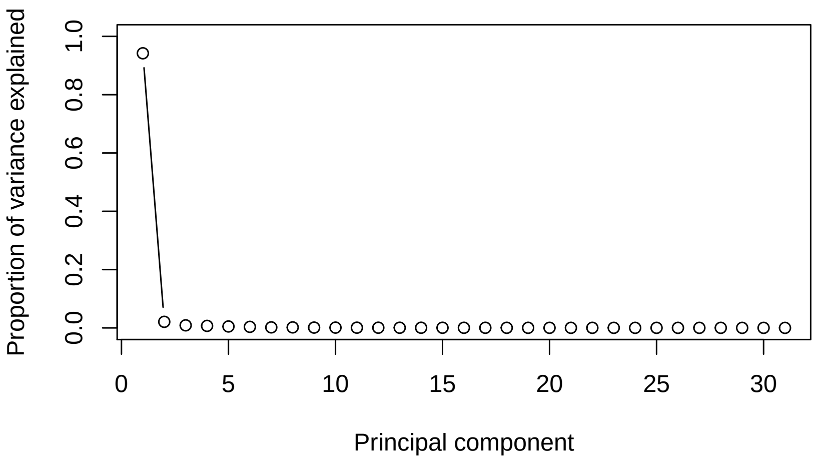

Figure S4: Screeplot describing proportion of variances explained by each principal component of the PCA completed on the estimated expression abundances of *E. salsugineum* ecotypes subjected to a progressive drought. Expression estimates were calculated from RNASeq data.

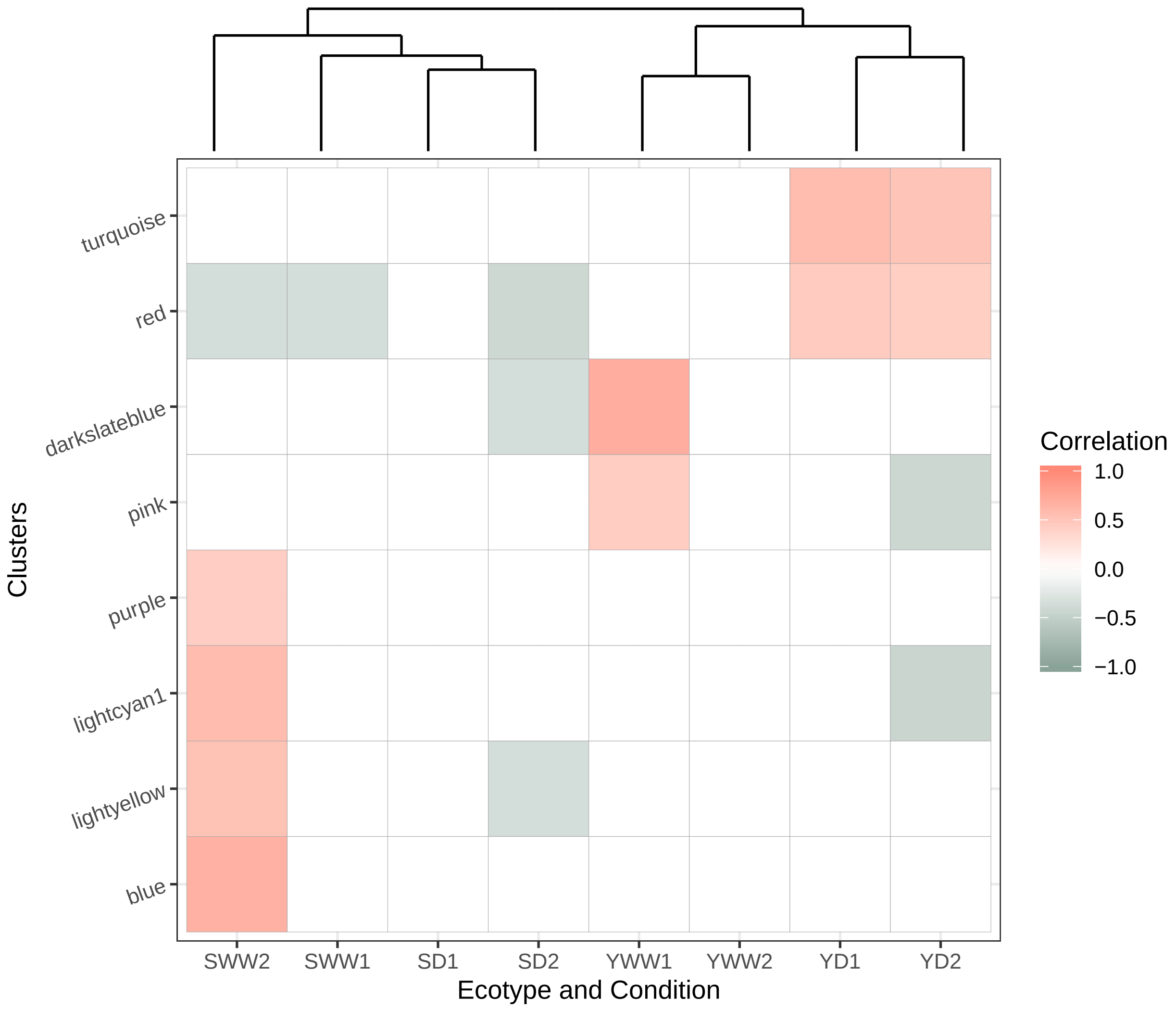

Figure S5: WGCNA cluster heatmap illustrating correlations of selected cluster eigengenes (Y axis) to ecotype and progressive drought conditions (X axis). Positive correlations are coloured in coral, negative correlations are coloured in slate and non-significant correlations are represented in white. Significance was defined as p < 0.05 after false discovery rate (FDR) adjustment. Samples were clustered by hierarchical clustering of all correlations, not only the select clusters.

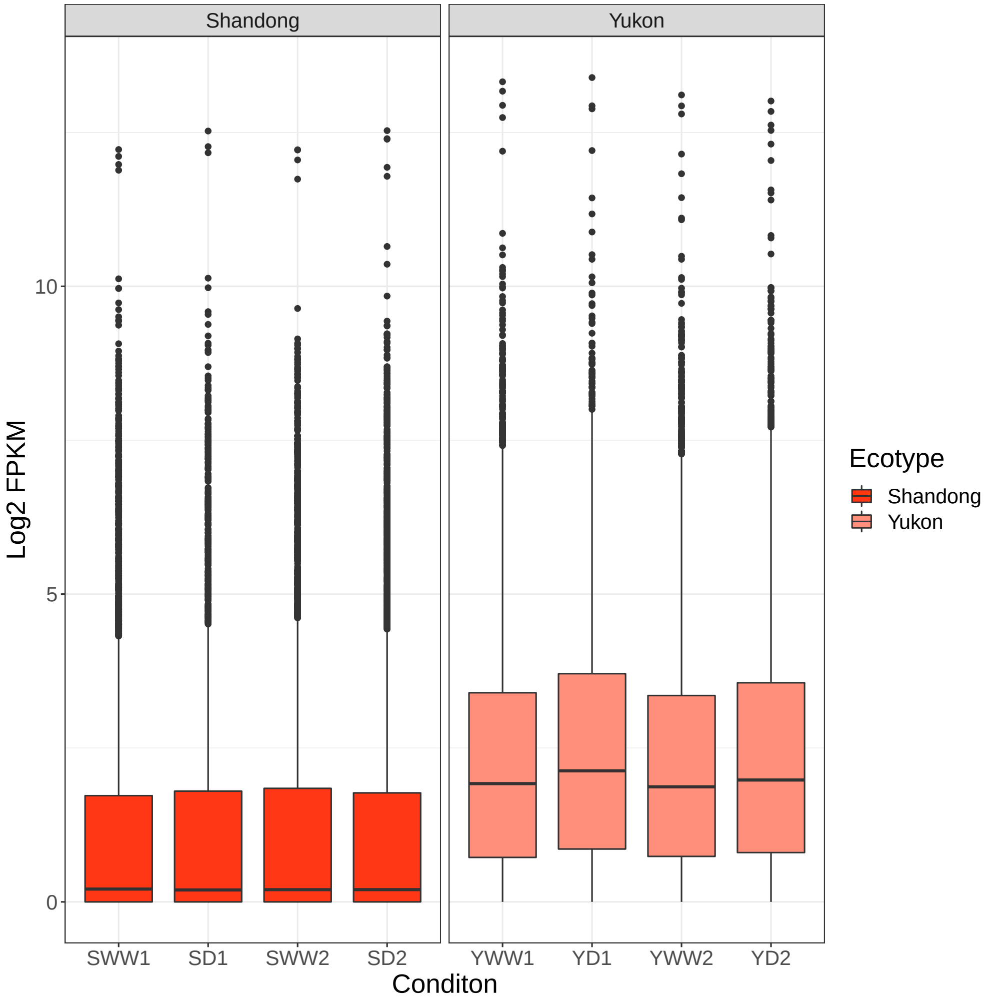

Figure S6: Boxplot of $\log_2$ FPKM values of genes in the "red" cluster with lines representing median values. Outliers appear as points.

References

M. J. Champigny, W. W. Sung, V. Catana, R. Salwan, P. S. Summers, S. A. Dudley, N. J. Provart, R. K. Cameron, G. B. Golding, and E. A. Weretilnyk. RNA-Seq effectively monitors gene expression in *Eutrema salsugineum* plants growing in an extreme natural habitat and in controlled growth cabinet conditions. *BMC Genomics*, 14:578, 2013. doi: 10.1186/1471-2164-14-578.

Mitchell J. R. MacLeod, Jeff Dedrick, Claire Ashton, Wilson W. L. Sung, Marc J. Champigny, and Elizabeth A. Weretilnyk. Exposure of two *Eutrema salsugineum* (*Thellungiella salsuginea*) accessions to water deficits reveals different coping strategies in response to drought. *Physiologia Plantarum*, 155(3):267–280, 2015. doi: https://doi.org/10.1111/ppl.12316.

Jie Yin, Michael J Gosney, Brian P Dilkes, and Michael V Mickelbart. Dark period transcriptomic and metabolic profiling of two diverse *Eutrema salsugineum* accessions. *Plant Direct*, 2(2):e00032, 2018. doi: https://doi.org/10.1002/pld3.32.
